# Network pharmacology study of *Curcuma longa L.*: potential target proteins and their functional enrichment analysis

**DOI:** 10.1186/s13104-020-05301-0

**Published:** 2020-10-07

**Authors:** Sangeeta Kumari, Hosahalli S. Subramanya

**Affiliations:** Institute of Bioinformatics and Applied Biotechnology, Biotech Park, Electronics City Phase 1, Bengaluru, 560100 Karnataka India

**Keywords:** Markov clustering, Protein clusters, Centrality measure, Synergistic mechanism

## Abstract

**Objective:**

This study’s primary goal is unraveling the mechanism of action of bioactives of *Curcuma longa L.* at the molecular level using protein–protein interaction network.

**Results:**

We used target proteins to create protein–protein interaction network (PPIN) and identified significant node and edge attributes of PPIN. We identified the cluster of proteins in the PPIN, which were used to identify enriched pathways. We identified closeness centrality and jaccard score as most important node and edge attribute of the PPIN respectively. The enriched pathways of various clusters were overlapped suggesting synergistic mechanism of action. The three pathways found to be common among three clusters were Gonadotropin-releasing hormone receptor pathway, Endothelin signaling pathway, and Inflammation mediated by chemokine and cytokine signaling pathway.

## Introduction

The *Curcuma longa L.* has been studied for antiinflammatory and anticancer effects [1]. The exact mechanism remains largely unexplored. Bioactives have shown the multi-components and multi-targets effect by using protein–protein interaction network (PPIN). A target protein usually carries out a typical function by regulating other molecules; thus, the study of PPIN helps to understand relationship between target proteins and other interacting proteins in a systematic way.. Earlier study has shown that the target proteins indeed have some special topological features that are significantly different than the normal proteins [2]. Thus, we decided to do a comparison study of a true PPIN and a false PPIN to identify discriminating topological attributes. Further, we used those attributes to select importantnodes and edges in the PPIN.

## Main text

### Methods

The four bioactive compounds namely curcumin, Desmethoxycurcumin, Bisdemethoxycurcumin and Turmerone of *C. longa* were studied. We used similarity ensemble approach (http://sea.bkslab.org/) to identify the potential target proteins of all these four bioactive compounds [3]. Further, we queried the target proteins to StringDB (human protein interaction database) to retrieve all the listed interactions involving the target proteins. A small set of target proteins (TP) was found to have interaction (biological or physiochemical) with many other interacting proteins (IP). We used NetworkX library in python to build and study the true and false PPIN.

To create the true PPIN, we created an undirected graph having edge indicating the interaction between the TP and IP as obtained from StringDB.

The false PPIN was created by forming edges belonging to all the non-existent interactions for TP and IP.

We used Markov-clustering library in python to identify protein clusters in the network. We used statistical overrepresentation test of PANTHER pathways (http://pantherdb.org/) to identify significant pathways (using human reference genome) associated with the each cluster of the network.

#### Calculation

We created true PPIN network using TP and IP as nodes and StringDB interactions as edges. For false PPIN, TP and IP were used as nodes with the non-existent interaction as edge. We calculated edge property of both the networks using four link prediction algorithms; jaccard_score, preferential_attachment score, common_neighbors score, and resource_allocation_index score, using NetworkX library in python. Further, we calculated node topological property of both the networks; such as, degree, eigenvector centrality, betweenness centrality, closeness centrality, local clustering coefficient, eccentricity values.

We identified best edge attribute and node attribute by using statistical analysis. The codes are available at Github.

### Results and discussion

#### Similarity Ensemble Approach

**Fig. 1:**
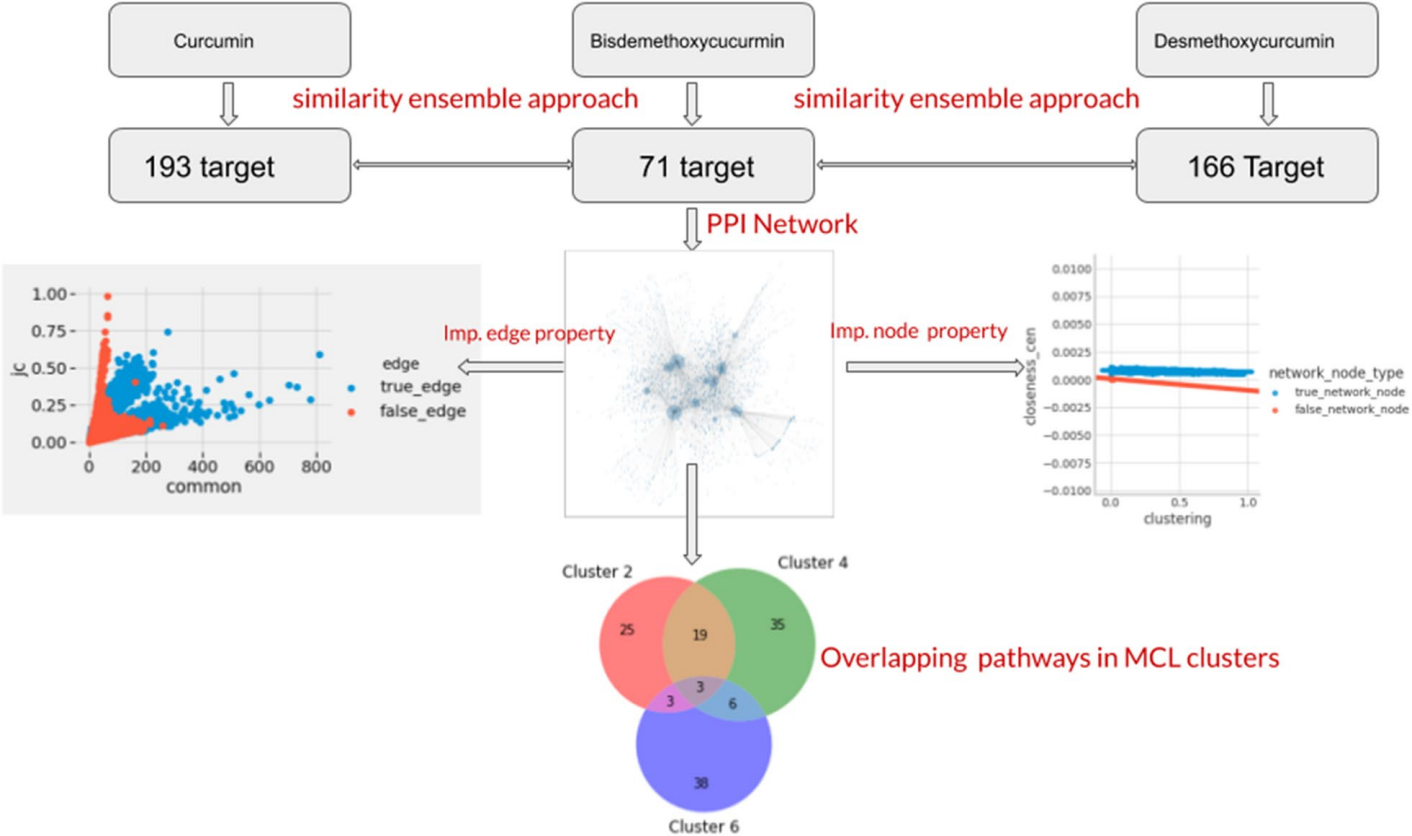
Schematic representation of workflow used in this study

The chemical-centric method can exploit the pharmacological relationships among protein targets in addition to their biological [4]. The target molecule Curcumin was queried and we found 193 human target proteins associated with it. Desmethoxycurcumin was mapped to 166 human target proteins, Bisdemethoxycurcumin identified to have 71 human target proteins and Turmerone was associated with 2 target proteins. After removing overlapped target proteins, we had 219 unique target proteins for further PPIN study (Fig. 1).

#### Network formation and property

We downloaded Human protein interaction data (scored links between proteins) from String DB and retrieved all the interaction in which any of the 219 target proteins were involved. This has led to 208,125 interactions having interaction score from 150 to 999. We removed edges having score below 300 which gave a total of 58,482 interactions as edgeand 11,979 (TP + IP) proteins as nodes. The nodes were comprised of TP (219) and IP (11,760).

#### Biological interactions network (True PPIN) vs. False interaction network (false PPIN)

To understand the network property of both the networks (true PPIN and false PPIN), we calculated the four different edge attributes (scores) using link prediction algorithms. Thus, we calculate the score value for each edge in both the networks. To calculate the score value, we used different algorithms implemented in the Networkx library in python. The calculated scores were namely 1) preferential attachment score: Preferential attachment algorithm shows that the more connected a node is, the more likely it is to receive new links. Thus an edge which connects two nodes which themselves are highly connected to other nodes (by an edge) will have higher edge score value. 2)common neighbors score: Common neighbors algorithm captures the idea that two strangers who have a friend in common are more likely to be introduced than those who don’t have any friends in common. Thus, an edge which connects two nodes which are having higher number of common connection (other nodes which they are connected to) will have higher value of edge score. 3) jaccard score: jaccard score is a measure used to compute the closeness of nodes based on their shared neighbors and their degree values. The higher jaccard score value for an edge (connecting two nodes) shows that the two nodes are having higher number of common connection but themselves are not highly connected to other nodes. and 4) resource allocation score: resource allocation score is a measure used to compute the closeness of nodes based on their shared neighbors and the degree value of that shared neighbor nodes. The higher resource allocation score value for an edge (connecting two nodes) shows that the two nodes are having higher number of common connections and those common connections are not highly connected to the other nodes. To calculate these edge score using above mentioned four link prediction algorithms.

For true PPIN, we calculated the correlation coefficient of score values of edge attributes along with interaction score obtained from StringDB using Pearson correlation coefficient. We found a poor correlation between interaction score against each of the topological edge attributes. The obtained correlation coefficient values ranges from 0.076 to 0.31. Thus, none of the topological edge attributes resembled the biological interactions between two protein nodes. Further, we performed the significance testing of edge attributes belonging to the two groups; true PPIN and false PPIN. The most significant edge attribute between the two groups obtained by *t* test was jaccard score. The t-tests results are uploaded on the Github as folder named Edge_attributes_hypothesis_testing.

#### Difference in centrality measures

Further, we studied the node attributes of these two networks, and calculated different types centrality measures. We calculated the degree, closeness centrality, Eigenvector Centrality, betweenness Centrality, Local Clustering Coefficient, Eccentricity.

We calculated the correlation coefficient of all the centrality measures for the true PPIN and false PPIN. For true PPIN, we found the very strong correlation between degree and betweenness centrality (0.95) which shows that nodes with high degree control the information flow in the network by being present in shortest paths in PPIN and may contribute to multiple pathways.

For false PPIN, we found the very strong correlation between degree and eigenvector centrality (0.93) but a poor correlation between degree and betweenness centrality (0.56). This showed that the unlike true PPIN, high degree nodes do not control the information flow in the network.

Further, we used the machine learning algorithm such as logistic regression and random forest to select best classifier node attributes to differentiate between the true PPIN and false PPIN. The closeness centrality was identified as a best classifier. For true PPIN, nodes have relatively higher values for closeness centrality.

By using our findings, we removed the insignificant edges and nodes from true PPIN and made it sparse. We removed edges having jaccard score value above 75 percentile of true PPIN. We also removed the nodes that had closeness centrality value less than the 25 percentiles in true PPIN. This yielded a resulting network of 1900 nodes and 4637 edges (Fig 1).

#### Protein cluster identification

**Fig. 2:**
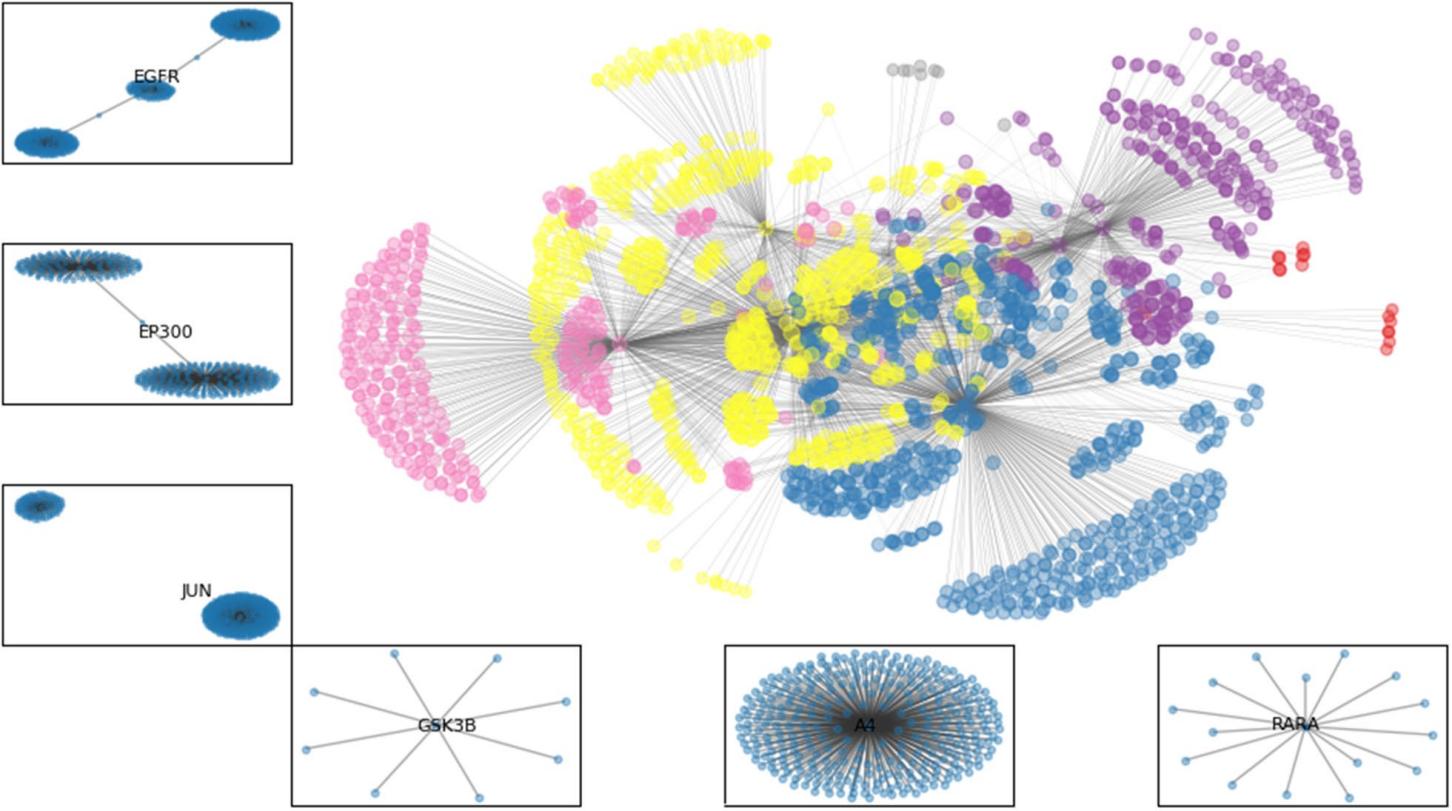
Module identification of PPI network using MCL clustering and target protein of each cluster

We used Markov cluster (MCL) algorithm for protein cluster identification. MCL algorithm is particularly noise-tolerant as well as effective in identifying high-quality protein clusters [5]. MCL is unsupervised cluster algorithm for graphs based on manipulation of transition probabilities to identify protein clusters. Protein clusters are generally highly overlapped but MCL is hard clustering algorithm and proteins are non-overlapping. The fundamental concept of identifying protein clusters is that a pair of proteins interacting with each other has higher probability of sharing the same function (pathway) than two proteins not interacting with each other. The MCL algorithm identified 6 clusters within true PPIN (Fig. 2).

#### Pathway enrichment analysis

Target identification and synergistic interaction among multiple target is important unravel the pharmacological mechanism of action of bioactives. Target proteins belonging to each cluster were searched into Gene Ontology database (http://pantherdb.org/webservices/go/overrep.jsp). We uploaded the protein list of each cluster, we selected the option of statistical overrepresentation test.

A detailed table showing the cluster number, their TP, IP and pathways is uploaded on the Github page as cluster_proteins_pathways.xlsx. we can conclude that the three cluster involved in the significant number of pathways are cluster number 2, 4, and 5 contributing to 25, 35 and 38 pathways respectively. Three pathways were overlapped among these three cluster. These pathways were Gonadotropin-releasing hormone receptor pathway, Endothelin signaling pathway, and Inflammation mediated by chemokine and cytokine signaling pathway. Earlier studies [6] showed the connection between presence of Gonadotropin-releasing hormone receptor in extra-pituitary tissues and progression of some cancers which gives indirect evidence to the anticancer activity of the *C. longa.*

## Limitations

- Experimental study is not included in the which was ideal to assess pathways enrichment.
- Lack of complete information about target proteins and theirinteraction.

## Data Availability

The datasets generated during the current study are available in the Network_Pharmacology repository, https://github.com/sangeeta97/Network_Pharmacology.

## Abbreviations

PPIN: Protein-protein interaction network
PPI: Protein-protein interaction
MCL: Markov cluster algorithm
TP: Target proteins
IP: Interacting proteins

## References

[1] Gan Y, Zheng S, Baak JP (2015). Prediction of the anti-inflammatory mechanisms of curcumin by module-based protein interaction network analysis. Acta Pharm Sin B..

[2] Vallabhajosyula RR, Chakravarti D, Lutfeali S, Ray A, Raval A (2009). Identifying hubs in protein interaction networks. PLoS ONE.

[3] Keiser MJ, Roth BL, Armbruster BN, Ernsberger P, Irwin JJ, Shoichet BK (2007). Relating protein pharmacology by ligand chemistry. Nat Biotechnol.

[4] Wang Z, Liang L, Yin Z, Lin J. Improving chemical similarity ensemble approach in target prediction. *J Cheminform*. 2016;8:20. Published 2016 Apr 23. 10.1186/s13321-016-0130-x. 10.1186/s13321-016-0130-x PMC4842302 27110288

[5] Enright AJ, Van Dongen S, Ouzounis CA (2002). An efficient algorithm for large-scale detection of protein families. Nucleic Acids Res.

[6] Lu M, Zhu J, Ling Y, Shi W, Zhang C, Wu H (2015). The lower expression of gonadotropin-releasing hormone receptor associated with poor prognosis in gastric cancer. Int J Clin Exp Med..

